# Vitamin D supplementation in older people (VDOP): Study protocol for a randomised controlled intervention trial with monthly oral dosing with 12,000 IU, 24,000 IU or 48,000 IU of vitamin D_3_

**DOI:** 10.1186/1745-6215-14-299

**Published:** 2013-09-17

**Authors:** Inez Schoenmakers, Roger M Francis, Elaine McColl, Thomas Chadwick, Gail R Goldberg, Christine Harle, Alison Yarnall, Jennifer Wilkinson, Jennie Parker, Ann Prentice, Terence Aspray

**Affiliations:** 1 MRC Human Nutrition Research, Elsie Widdowson Laboratory, Fulbourn Road, Cambridge CB1 9NL, UK; 2 Institute for Ageing and Health, Newcastle University, Campus for Ageing and Vitality Nuns Moor, Newcastle upon Tyne NE4 5PL, UK; 3 Newcastle Clinical Trials Unit, The Medical School, 4th Floor, William Leech Building Framlington Place, Newcastle upon Tyne NE2 4HH, UK; 4 Institute of Health and Society, Newcastle University, Baddiley-Clark Building Richardson Road, Newcastle upon Tyne NE2 4 AX, UK; 5 Bone Clinic Musculoskeletal Unit, Freeman Hospital, Newcastle upon Tyne NE7 7DN, UK

**Keywords:** Vitamin D supplementation trial, Bone mineral density, 25 hydroxy vitamin D, Parathyroid hormone, Bone markers, Older people

## Abstract

The randomised, double blind intervention trial ‘Optimising Vitamin D Status in Older People’ (VDOP) will test the effect of three oral dosages of vitamin D given for one year on bone mineral density (BMD) and biochemical markers of vitamin D metabolism, bone turnover and safety in older people. VDOP is funded by Arthritis Research UK, supported through Newcastle University and MRC Human Nutrition Research and sponsored by the Newcastle upon Tyne Hospitals NHS Foundation Trust.^a^

**Background:**

Vitamin D insufficiency is common in older people and may lead to secondary hyperparathyroidism, bone loss, impairment of muscle function and increased risk of falls and fractures. Vitamin D supplementation trials have yielded conflicting results with regard to decreasing rates of bone loss, falls and fractures and the optimal plasma concentration of 25 hydroxy vitamin D (25OHD) for skeletal health remains unclear.

**Method/design:**

Older (≥70 years) community dwelling men and women are recruited through General Practices in Northern England and 375 participants are randomised to take 12,000 international units (IU), 24,000 IU or 48,000 IU of vitamin D_3_ orally each month for one year starting in the winter or early spring. Hip BMD and anthropometry are measured at baseline and 12 months. Fasting blood samples are collected at baseline and three-month intervals for the measurement of plasma 25OHD, parathyroid hormone (PTH), biochemical markers of bone turnover and biochemistry to assess the dose–response and safety of supplementation. Questionnaire data include falls, fractures, quality of life, adverse events and outcomes, compliance, dietary calcium intake and sunshine exposure.

**Discussion:**

This is the first integrated vitamin D supplementation trial in older men and women using a range of doses given at monthly intervals to assess BMD, plasma 25OHD, PTH and biochemical markers of bone turnover and safety, quality of life and physical performance. We aim to investigate the vitamin D supplementation and plasma 25OHD concentration required to maintain bone health and to develop a set of biochemical markers that reflects the effect of vitamin D on bone. This will aid future studies investigating the effect of vitamin D supplementation on fracture risk.

#ISRCTN 35648481 (assigned 16 August 2012), EudraCT 2011-004890-10.

## Background

### Vitamin D status and musculoskeletal health

Vitamin D insufficiency is common in older people and may lead to increased bone resorption, bone loss, impairment of muscle function and an increased risk of falls and fractures. The results of clinical trials assessing the effect of vitamin D supplementation on bone loss, falls and fractures have yielded conflicting results and the blood concentration of 25 hydroxy vitamin D (25OHD) required to maintain optimal skeletal health remains unclear. Unfortunately, there is no universal consensus on the optimal way of assessing vitamin D status or on what constitutes vitamin D repletion at different ages throughout life [1].

Vitamin D status is currently assessed by measurement of plasma 25OHD concentration. In the UK, vitamin D deficiency has been defined as a plasma 25OHD less than 25 nmol/L, the upper end of the range at which vitamin D deficiency osteomalacia and rickets have been observed [2,3]. Recent epidemiological and cross-sectional studies have shown that 25OHD concentrations above 25 nmol/L may also be associated with secondary hyperparathyroidism, higher bone resorption, bone loss, impaired muscle function and a greater risk of falls and fragility fractures [4-8] when compared to concentrations of 40 to 75 nmol/L and above.

### Vitamin D status and requirements

A plasma 25OHD concentration of 40 to 50 nmol/L has been suggested as a cut-off for vitamin D insufficiency [2,9-11]. However, some experts have advocated higher concentrations of 25OHD (75 to 150 nmol/L) for the maintenance of bone health and other non-skeletal benefits [12-17]. Few individuals in the UK achieve these concentrations of 25OHD in winter and spring, when more than 80% of 45-year-old men and women have a plasma 25OHD concentration less than 75 nmol/L [18-20]. Vitamin D status is poorer in older people, where approximately 12% of people living in private households and 30% of care home residents have a plasma 25OHD concentration below 25 nmol/L throughout the year, reflecting their reduced exposure to sunlight, low dietary intake of vitamin D and potentially their higher expenditure of vitamin D [14,18,21].

The dietary Reference Nutrient Intake (RNI) for vitamin D in the UK for people over 65 years is currently 400 International Units (IU) (= 10 μg) per day. In the US, the Estimated Average Requirement (EAR) and Recommended Dietary Allowance (RDA) for those over 70 years of age is 600 (15 μg) and 800 IU (20 μg) per day, respectively [11,22]. It is recognised that supplementation is generally necessary to achieve these intakes [23]. Despite this advice, vitamin D supplementation is uncommon in older people, even in care home residents [18,24-26].

### Vitamin D supplementation and fracture risk

Several placebo-controlled intervention trials have examined the effect of vitamin D supplementation on fracture risk. Some meta-analyses suggest that vitamin D supplementation doses of 800 to 1000 IU daily decrease the incidence of falls and fractures in older women, whereas lower doses are ineffective [27-29]. In contrast, other meta-analyses, including the latest Cochrane Review [30], indicate that vitamin D supplementation does not decrease fracture risk, unless combined with calcium, when it reduces the risk of hip and other non-vertebral fractures in care home residents [31,32]. One randomised controlled trial of a single annual oral dose of 500,000 IU vitamin D showed an increase in falls and fractures, particularly in the first three months after administration [33]. Other studies using 300,000 or 600,000 IU per annum have shown mixed results: 100,000 IU administered at four-month intervals was associated with a decrease in fracture risk, whereas 300,000 IU of vitamin D_2_ by i.m. injection or 150,000 IU administered orally every three months was associated with no benefit [34-36]. These results suggest that not only the dose but also the frequency of administration may determine the risk and benefit for falls and fractures. The mechanisms of this association remain unclear and are likely to be multi-factorial.

The evidence for the effects of vitamin D supplementation on BMD or the prevention of bone loss in older people is conflicting [37,38]. The differences in the reported efficacy of vitamin D may be because the dose of vitamin D in several trials may have been too low or compliance and persistence with supplementation may have been poor, resulting in a failure to achieve a high enough plasma 25OHD to reduce bone loss and obtain an anti-fracture benefit [13,39-41]. Alternatively, the participants in some trials may have been vitamin D replete [41], even if their baseline plasma 25OHD concentrations were lower than those advocated by North American experts [12,15,16]. Moreover, differences in reported efficacy may have been due to differences in the study population, the dose of vitamin D, the route and frequency of administration and/or the use of different forms of vitamin D (vitamin D_2_ and vitamin D_3_).

Several studies have indicated that the achieved plasma 25OHD concentration, rather than the vitamin D dose given, predicts the effect on bone health [42]. However, measurement of plasma 25OHD, parathyroid hormone (PTH) and other potential markers of vitamin D status have only been performed in a small minority of participants in these intervention studies [41], limiting the ability to explore the relationship between plasma 25OHD concentrations achieved and bone loss and fracture prevention.

### Vitamin D status markers for bone health

Before performing further studies of the effect of vitamin D supplementation on the risk of major outcomes such as fragility fractures or cardiovascular disease [12,13,43,44], there is a need to understand better the relationship between vitamin D intake, plasma 25OHD and bone health in older people and to develop better functional markers of vitamin D status, reflecting vitamin D function at the level of the target organ [3,45]. These functional markers will be instrumental in establishing clearer criteria for vitamin D sufficiency and insufficiency and help to clarify the optimal dose and frequency of vitamin D supplementation. Potential markers of vitamin D status have been reviewed [3]. Plasma PTH has been proposed as a functional marker, because an elevated concentration is a recognised risk factor for bone loss and fractures in older people [6-9,13,46-48], which can potentially be modified by vitamin D supplementation [49,50]. The circulating concentration of 25OHD below which PTH increases outside the normal range may be used to establish a threshold value for vitamin D insufficiency [1]. However, PTH varies within and between people at any given concentration of 25OHD [18,51], as PTH depends on many factors other than vitamin D status [52]. These include stage of life, ethnic background, dietary calcium and phosphate intake, time of day, renal function, serum magnesium, physical inactivity and medication use [3,14,53-55].

### Aim

The Optimising Vitamin D Status in Older People (VDOP) trial aims to examine the relationship between vitamin D supplementation at a range of doses and the change in bone mineral density (BMD) in older people living in private households in the North East of England. Vitamin D_3_ supplementation is given orally each month, for a year (12,000 IU/month, 24,000 IU/month or 48,000 IU/month, equivalent to 400 IU/day, 800 IU/day and 1,600 IU/day, respectively). We will explore the relationship between the change in BMD and the achieved plasma 25OHD and PTH over 12 months. As BMD is the result of the balance between bone formation and resorption, the effect of the supplement on the change in markers of bone turnover will be explored. Plasma C-terminal telopeptide (CTX), bone specific alkaline phosphatase (bone ALP) and N-terminal propeptide of procollagen type I (P1NP) will be measured, as it has been suggested that these may be the best surrogate bone turnover markers for the effect of pharmacological intervention on fracture incidence [56]. We will also investigate the effect of duration of treatment and season on the dose–response.

### Rationale for dosage and dosing regimen

Two of the dosages applied in this trial correspond to the current Scientific Advisory Committee on Nutrition (SACN) RNI (400 IU/day) and the Institute of Medicine (IOM) RDA (800 IU/day) for this age group [2,11]. The highest dose is twice the IOM RDA and well below the IOM Tolerable Upper Intake Level (TUIL) defined by the IOM (4,000 IU/day) [11].

This trial uses a monthly dosage scheme as this is expected to result in improved compliance compared to daily or weekly dosages [57]. Compliance will be encouraged through administration of the dose under direct supervision on four occasions and taking the other doses will be prompted by telephone calls by staff from the research centre.

## Methods/design

### Study design

The VDOP trial is a single centre parallel group, randomised, double blind interventional trial testing the effects of three dosages of oral vitamin D_3_ given each month to men and women 70 years old and older for a year. The primary outcome is the change in BMD at the hip (total hip BMD) from baseline to 12 months post intervention.

### Recruitment

Community dwelling men and women 70-years-old and older, are recruited through General Practices (GP) in Tyneside, Wearside and Northumberland, participating in the Northern and Yorkshire Primary Care Research Network (PCRN-NY). Potentially eligible participants are identified from GP practice registers, after initial screening against inclusion and exclusion criteria on electronic medical records. An invitation letter is sent to potential participants, including details of the study and a pre-paid response slip. Those expressing an interest are contacted by the research team, further screening performed to ensure that the participant is eligible and an invitation to participate is then offered, after any discussions the potential participant might wish. If still interested, an appointment is made for the first study visit, when written informed consent is obtained, and a blood sample is collected to perform the final assessment of eligibility (screening for biochemical exclusion criteria: see below). If eligible, baseline visit measurements and questionnaires are completed.

### Inclusion criteria

Inclusion criteria are as follows:

1. Ambulatory, community dwelling men and women 70 years old and older

2. Individuals capable of giving informed consent on their own behalf

3. Individuals willing to attend the Study Centre (The Clinical Ageing Research Unit (CARU)) on six occasions and to be contacted by telephone at monthly intervals between study visits over twelve months

### Exclusion criteria

Exclusion criteria are as follows:

1. Current antiresorptive or anabolic treatment for osteoporosis

2. Treatment with bisphosphonates for osteoporosis in the past two years

3. Current supplement use of vitamin D (>400 IU/day) or calcium (>500 mg/day) (including use of over-the-counter preparations)

4. Fragility fracture in the previous six months

5. Known primary hyperparathyroidism

6. History of renal stones

7. Hip replacement

Biochemical:

1. Hypercalcaemia (albumin-adjusted serum calcium >2.60 mmol/L)

2. Renal impairment (Stage 4 or 5 Chronic Kidney Disease: estimated glomerular filtration rate (eGFR) below 30 ml/min/1.73m^2^ calculated using the modification of diet in renal disease (MDRD) formula.

There will be no limits to concomitant care or medication use other than defined in the inclusion and exclusion criteria. Participation in the VDOP trial is discontinued when during the course of the trial conditions develop where vitamin D supplementation is contra-indicated.

### Randomisation, Investigational Medicinal Product (IMP) and IMP administration

Participants are randomised using a computerised system in a 1:1:1 ratio to three treatment arms (12,000 IU, 24,000 IU or 48,000 IU vitamin D_3_ once monthly for 12 months) by a statistician within the Newcastle Clinical Trials Unit (CTU) not otherwise engaged in the study. Permuted blocks of variable length are used to reduce the risk of breach of concealment of allocation. Allocation lists are provided to Apotek Produktion & Laboratorier AB (APL; Stockholm, Sweden) which produces numbered packs of the Investigational Medicinal Product (IMP) per participant for shipping to the Newcastle upon Tyne Hospitals NHS Foundation Trust pharmacy for dispensing. The allocation is not stratified and allocation status is kept concealed from participant and research staff unless code break is required for safety reasons.

The IMP is composed of vitamin D_3_ dissolved in Miglyol as Vigantol® (Merck Sereno GmbH, Darmstadt Germany). One ml of Vigantol® solution contains 0.5 mg cholecalciferol, equivalent to 20,000 IU vitamin D_3_. Blinding of Vigantol® oil across the three doses (12,000 IU, 24,000 IU and 48,000 IU) is achieved by further dilution, using Miglyol® 812 oil (Sasol; The Warner Graham Company, Cockeysville, MD USA) and the stabilising agent butylhydroxianisole, both free of vitamin D_3._ For the highest dose, only the stabilising agent is added. All three doses of vitamin D are dispensed in 10 ml amber glass bottles (target fill volume of 3 ml including 0.6 ml overage).

MODEPHARMA (London, UK) is responsible for arranging the manufacture of the IMP and project management and assistance relating to the IMP. The formulation, manufacture and release of the IMP is undertaken by APL, a Swedish clinical supplies company licensed for the manufacture and release of IMPs for clinical trial use in the EU.

The IMP is administered orally under direct supervision of a research nurse at baseline and at the third, sixth and ninth month study visits. At the end of each study visit, participants are given two bottles of the study IMP for the two subsequent months before their next scheduled visit to CARU, together with written instructions on how to take the IMP. On months when the participant is not attending the clinic, they are contacted by telephone, to remind them to take the IMP. Participants are directed to return all IMP bottles in their original packaging to the research team. Researchers return these to the trial pharmacist and all returned or unused study IMPs are documented in the Pharmacy File and then destroyed according to Pharmacy Standard Operating Procedures.

### Measurements

An overview of the timing of measurements, sample collections and data collection is given in Table 1. Total hip BMD is measured using Dual-Energy X-ray Absorptiometry (DXA) (i-Lunar DXa, GE Healthcare, Madison Wisconsin USA) at baseline and 12 months. The densitometer is calibrated daily prior to participant scanning. The total radiation dose (0.04 mSv) is equivalent to having a chest X-ray. Height and weight, demispan, waist-hip ratio and the Timed Up and Go (TU&G) test are determined [58] and the participant’s medical history (including medication and supplement use) reviewed. A standardised physical exam including the assessment of vital signs, blood pressure, and questionnaires assessing bone pain, fracture risk and falls history are performed (adapted from standard clinical care questions and the Fracture Risk Assessment (FRAX) tool [59]). Blood and timed urine samples are collected after an overnight fast between 8:30 and 11:30 AM. After venipuncture, participants are offered breakfast. Questionnaires are used to collect data on demographics, quality of life (WHOQol-BREF UK version and UK WHOQOL-old; [60,61]), dietary calcium and vitamin D intake (adapted from Calquest; [62]) and sunshine exposure in the UK and during overseas travel (adapted from [63]). Participants are also asked to keep a three-month prospective falls diary. At each visit, information is collected on participant-reported adverse events (AEs), serious adverse events (SAEs) and suspected unexpected serious adverse reactions (SUSARs). In addition, at every visit, safety is assessed specifically by performing full blood count, serum albumin adjusted calcium, creatinine and liver function. Information on clinical fractures during the study is recorded as a safety measure.

**Table 1:** Schedule of study procedures for VDOP

		**Phone call 0**	**Visit 0**	**Visit 1**	**Phone call 1**	**Phone call 2**	**Visit 2**	**Phone call 3**	**Phone call 4**	**Visit 3**	**Phone call 5**	**Phone call 6**	**Visit 4**	**Phone call 7**	**Phone call 8**	**Visit 5**
	Pre- Screening	Pre- Screening	Screening	Baseline visit	FUP^a^ 1	FUP^a^ 2	FUP^a^ 3	FUP^a^ 4	FUP^a^ 5	FUP^a^ 6	FUP^a^ 7	FUP^a^ 8	FUP^a^ 9	FUP^a^ 10	FUP^a^ 11	Final visit
Venue	PIC^a^	Home	CARU^a^	CARU^a^	Home	Home	CARU^a^	Home	Home	CARU^a^	Home	Home	CARU^a^	Home	Home	CARU^a^
Month	−1	0	0	**0**	1	2	**3**	4	5	**6**	7	8	**9**	10	11	**12**
Identification	X															
Pre-Screening		X														
Informed consent			X													
Venipuncture (screening)			X													
Randomisation			X													
Medical history, Demispan,, falls history and fracture risk				X												
Demographics				X												
TU&G^a^ test grip strength				X												X
DXA (total hip) weight height				X												X
Venipuncture (safety and biochemistry)				X			X			X			X			X
Urine sample				X			X			X			X			X
Physical exam and vital signs				X			X			X			X			X
Quality of Life			X				X			X			X			X
CalQuest			X							X						X
Sunshine exposure			X				X			X			X			X
Falls diaries				----------	----------	-------➔	X	---------	-------➔	X	---------	-------➔	X	---------	-------➔	X
Administer treatment				X	X	X	X	X	X	X	X	X	X	X	X	
Check drug adherence					X	X	X	X	X	X	X	X	X	X	X	X

^*a*^*CARU* Clinical Ageing Research Unit, *FUP* follow-up visit, *PIC* Patient Identification Centre, *TU&G* Time up and Go.

### Biochemical analyses

Blood samples are collected into tubes containing ethylenediaminetetraacetic acid (EDTA), lithium heparin (LH) or serum-separation gel (BD Diagnostics, Oxford, UK) and one EDTA and one LH tube is immediately placed on ice. Samples for clinical biochemistry and safety measurements are sent to Newcastle upon Tyne hospitals NHS Foundation Trust (NUTH) laboratories and analysed immediately. These include eGFR, full blood count (FBC), urea and electrolytes (U&E) and liver function tests (LFT) which include: serum sodium, potassium, urea, total protein, total bilirubin, alanine transaminase, calcium, phosphate, magnesium, albumin, alkaline phosphatase (ALP) and creatinine. The remaining blood samples are separated within 30 minutes of collection in a refrigerated centrifuge at 1,800 g for 20 minutes and stored at −80°C prior to analysis at MRC Human Nutrition Research, Cambridge, UK. Blood pellets are stored at −20°C for potential genetic analyses. Collected urine is mixed thoroughly and aliquots are analysed for calcium, phosphate and creatinine (Cr) immediately at NUTH laboratories.

All assays are performed in duplicate with the exception of PTH. EDTA plasma is used for analysis of PTH by immunoassay (Immulite, Siemens Healthcare Diagnostics Ltd, Camberley, UK). LH plasma is used for the measurement of 25OHD, BAP (both DiaSorin-Liaison, Stillwater, Mn, USA) and CTX (IDS Ltd, Tyne and Wear, UK) according to standardised protocols [64]). Assay performance is monitored using kit and in-house controls and under strict standardisation according to ISO 9001:2000. Quality assurance of 25OHD and PTH assays are performed as part of the Vitamin D External Quality Assessment Scheme (http://www.deqas.org), and the National External Quality Assessment Scheme (http://www.ukneqas.org.uk).

### Data entry and analyses of questionnaires

An electronic case report form (eCRF) is created for each participant using a standardised computerised system (InferMed’s MACRO) at CTU. Biochemistry data are generated electronically and collated in a standardised format. Dietary contents of calcium and vitamin D are derived from food tables. Quality checks of data entry and merging are performed.

### Sample size and power calculation

The sample size calculation was based on an unpaired two-group comparison of the absolute change in BMD at the hip within individual participants over the 12 months of the study. This illustrates the power for a primary comparison of interest between any two chosen arms in the three-arm design of this study; given the choices detailed below, the calculations would be identical for any selected pair of arms. Absolute change is used, rather than percentage change, as it is statistically more efficient [65].

Data from a population based study [50] showed that standard deviation (SD) in absolute change of BMD at the hip over 12 months in women 60 to 70 years old, living in Aberdeen, is between 0.012 and 0.015 after supplementation with a placebo or two vitamin D dosages (0, 400 IU and 1,000 IU). As this SD is relatively stable in value but does not include values for two of our three dosage levels, the conservative choice was made to use the largest SD estimate and to apply the two-sample t-test, a two-sided significance level of 0.05 and a power of 80% in the calculations. Aiming to be able to detect a difference of 0.006 g/cm^2^ (equivalent to a moderate standardised effect size of 0.4) between two arms, 100 participants per arm would be needed. Making an allowance for 20% attrition at the 12 month follow-up results in 125 participants per arm (375 in total) being required. This sample size would give 90% power to detect a difference of 0.007 g/cm^2^ between a pair of arms. We believe that a difference in BMD of the order of 0.006 g/cm^2^ against the background of the anticipated yearly decrease in BMD in an untreated population would be clinically significant [50].

### Statistical analyses

The primary outcome of this study is the 12 month change in BMD at the hip (total hip BMD).

The secondary outcome measures are the changes in plasma 25OHD concentration and PTH, so as to explore their use as functional markers in relation to change in BMD and biochemical markers of bone turnover. Data are also being obtained on the proportion of participants in each treatment group who fall, the number of falls, quality of life and safety.

Analyses are performed according to a formal pre-planned documented analysis plan. Analysis will be carried out primarily on an intention-to-treat basis although other exploratory analyses may also be considered. There are no planned interim analyses.

Data with missing observations due to loss to follow-up will be examined to determine both the extent of missingness and whether it is missing at random or is selective. If data are missing to a sufficient extent, the use of appropriate multiple imputation techniques will be considered to allow for this in the analysis.

Analysis of the primary outcome measure (12 month change in BMD at the hip) will be performed using Analysis of Covariance (ANCOVA) to compare the response in the three treatment groups while allowing for the effects of covariates. Baseline BMD at the hip, PTH and 25OHD measures will be included in addition to measures of kidney function, BMI, gender and age. Interactions will also be explored. The inclusion of baseline values as covariates will also enable the examination of possible interactions between effects observed and these values.

A number of secondary analyses will also be undertaken. Alternative clinical indicators (such as change in plasma 25OHD, PTH, falls, physical performance, quality of life measures and other biochemical markers) will be compared between the three treatment groups, using similar techniques to those described above.

The relationship of PTH as a dependent variable with 25OHD and kidney function as independent variables will be examined using regression methods. Further exploratory regression models will explore the relationships of 25OHD and PTH with change in BMD and biochemical markers of bone turnover. The relationship between 25OHD and BMI will also be explored using similar regression methods.

The relationship of 25OHD at different time points as a dependent variable, with season and duration of supplementation as independent variables, will be examined using regression or repeated measures methods.

### Ethical considerations

This study has received a favourable opinion from the Sunderland Research Ethics Committee (REC, 12/NE/0050) and R and D approval from the Newcastle upon Tyne Hospitals NHS Foundation Trust (sponsor)*.* The conduct of this study is in accordance with the recommendations for physicians involved in research on human subjects adopted by the 18th World Medical Assembly, Helsinki 1964 and later revisions.

The study involves procedures that may be unfamiliar to participants and are not part of their standard medical care. These procedures are explained in information sheets provided to all potential participants prior to their screening visit and written informed consent is obtained prior to any study procedures. The consent form specifies the separate study components and includes an opt in/opt out section for genetic analyses and permission to be approached for potential affiliated studies. Participants who lack capacity to consent for themselves are excluded from this study. In the unlikely event that a participant loses capacity after they have provided consent to take part, their case will be reviewed by the Chief Investigator and the legal representative of the participant to decide whether to continue in the study.

The hip BMD DXA measurement involves a low dose of radiation (see measurements).

The latest Cochrane Review suggests that vitamin D supplementation may be associated with a small increased risk of gastrointestinal side effects, hypercalcaemia, renal stones and renal impairment, particularly when given with additional calcium or when active metabolites of vitamin D are used [32]. However, the doses to be used in this study are not generally associated with adverse events [11,22]. As we are using vitamin D alone, without additional calcium supplementation, we do not anticipate that this will pose a significant risk to participants. The highest dose of vitamin D_3_ used in this study is less than half the TUIL recently advised by the IOM [11,22].

### Reporting and evaluation of adverse events, serious adverse events and suspected unexpected serious adverse reactions

The development or progression of age-related medical conditions is anticipated to occur spontaneously during the study. On the basis of the latest Cochrane Review a small increased risk of side effects may be expected (see above). Any AEs, SAEs or SUSARs are verified against treatment notes/medical records and causality assessed using standard criteria according to Good Clinical Practice (GCP) guidelines [66]. All adverse events judged by the Trial Steering Committee (TSG) as having a reasonable suspected causal relationship to the IMP are considered to be adverse reactions and are reported to the sponsor, the Medicines and Healthcare products Regulatory Agency (MHRA) and REC in accordance with current legislation.

### Data protection

Data are handled, computerised and stored in accordance with the Data Protection Act 1998, guidelines for GCP [66,67] and local policy. Data collected on paper are entered on a secure validated clinical data management system (InferMed’s MACRO). No participant identifiable data will leave the study site. To preserve anonymity, any data or samples leaving the site will identify participants by a unique study identification code (linked in anonymised form). All identifiable study records and Investigator Site Files are kept at the site in a locked filing cabinet with restricted access and on encrypted and/or pass-word protected computers.

### Monitoring

The daily management lies with the Trial Management Group (TMG) which comprises Dr Terry Aspray (Chief Investigator (Chair)), Professor Roger Francis (Co-Investigator), Dr Tom Chadwick (Statistician), Dr Jennifer Wilkinson (Senior Trial Manager), Christine Harle (Trial Manager), Jennie Parker (Assistant Trial Manager), Ian Campbell (Assistant Director of Pharmacy) and Katie Argo (Clinical Trials Pharmacist). The UK CRC registered Newcastle Clinical Trials Unit (NCTU) has delegated authority from the sponsor for obtaining ethical and regulatory approvals, trial management and ensuring that the study is conducted in accordance with the principles of GCP and pharmacovigilance.

The TSG consists of the TMG and Professor David Reid (Independent Chair), Professor Elaine McColl (Co-investigator), Dr Gail Goldberg (Co-investigator), Dr Ann Prentice (Co-investigator), Dr Inez Schoenmakers (Co-investigator), Dr Elina Hypponen (Independent member), Professor Sue Lanham-New (Independent member) and two Lay members (Patient support group representatives).

The Data Monitoring and Ethics Committee (DMEC) comprises Professor Arduino Mangoni (Chair), Dr Nicola Peel and Dr Barbara Gregson (trialist and statistician).

### Exit strategy

Participants in this study will discontinue the study supplementation after 12 months of supplementation. They are then free to use over the counter supplements of vitamin D.

All clinically relevant findings will be reported to the participants’ GPs by the CI (Dr Terry Aspray) who will also offer advice on clinical management of bone-related health issues through the Freeman Hospital Musculoskeletal Unit.

## Discussion and potential impact

This study will provide clarification of the dose of vitamin D required to prevent bone loss in older people in the UK and will influence the formulation of dietary recommendations of vitamin D at the population level. This study is novel and clinically relevant, as it will compare the effect of three different doses of vitamin D on BMD and plasma 25OHD, PTH and biochemical markers of bone turnover in both men and women. This study evaluates the effects of vitamin D as mono-therapy rather than combined with calcium. The data complement current evidence on the balance of risks in using vitamin D with or without calcium supplementation [68]. The study also offers the opportunity to evaluate the impact of different dosages of vitamin D supplement on structural (DXA Scanning) and biochemical markers which allow a better definition of Vitamin D sufficiency for bone health and to determine an adequate vitamin D intake to maintain musculoskeletal function. We aim to develop a set of biochemical markers that reflect the effect of vitamin D intervention on bone. This will aid future larger studies investigating the effect of vitamin D supplementation on fracture risk.

Although the study is primarily powered to detect an effect of vitamin D supplementation on BMD, it will also provide data on the effect of vitamin D supplementation on other functional outcomes, such as quality of life, physical performance and falls. Further, safety data are collected and analysed for the effects of the different dosages, temporal trends and the cumulative dosage. In addition, this study will provide data on the effect of season on vitamin D status and other markers and improve knowledge of the role of monthly (rather than daily) dosing with vitamin D.

## Trial status

The VDOP study commenced recruitment in November 2012; recruitment is scheduled to be completed at end of June 2013, with follow-up for 12 months thereafter.

## Endnotes

^a^Freeman Hospital, Freeman Road, High Heaton, Newcastle-upon-Tyne, Tyne and Wear, NE7 7DN, United Kingdom.

## Abbreviations

25OHD: 25 hydroxy vitamin D; AE: Adverse event; ALP: Alkaline phosphatase; APL: Apotek produktion & laboratorier; AR: Adverse reaction; ARUK: Arthritis research UK; BMD: Bone mineral density; bALP: Bone alkaline phosphatase; CARU: Clinical ageing research unit; CTU: Clinical trials unit; CTX: C-terminal telopeptide Type 1; DMEC: Data monitoring & ethics committee; DXA: Dual energy X-ray absorptiometry; EAR: Estimated average requirement; e-CRF: Electronic case report form; EDTA: Ethylenediaminetetraacetic acid; eGFR: Estimated glomerular filtration rate; FBC: Full blood count; GCP: Good clinical practice; GP: General practitioner; IMP: Investigational medicinal product; IOM: Institute of medicine; IU: International unit; LFT: Liver function test; LH: Lithium heparin; MDRD: Modification of diet in renal disease; MHRA: Medicines and healthcare products regulatory agency; PCRN-NY: Primary care research network – Northern and Yorkshire; PIC: Patient identification centre; P1NP: Terminal propeptide of procollagen type I; PTH: Parathyroid hormone; QC: Quality control; QP: Qualified person; RDA: Recommended daily allowance; REC: Research ethics committee; RNI: Reference nutrient intake; SACN: Scientific advisory committee on nutrition; SAE: Serious adverse event; SAR: Serious adverse reaction; SUSAR: Suspected unexpected serious adverse reaction; TMG: Trial management group; TSG: Trial steering group; TU&G: Time up and go; TUIL: Tolerable upper intake level; U&E: Urea and electrolytes; Vitamin D2: Ergocalciferol; Vitamin D3: Cholecalciferol; VDOP: Optimising vitamin D status in older people; WHO QOL-OLD: WHO Quality of life measure for older people.

## Competing interests

The authors declare that they have no competing interests.

## Authors’ contributions

IS, RMF, EMcC, TC, GG, AP, TA designed the trial; IS, RMF, EMcC, TC, CH, AY, JW, JP and TA are responsible for the conduct of the trial; IS and TA drafted the paper; All authors read and approved the final manuscript.
